# An engineering viewpoint on biological robustness

**DOI:** 10.1186/s12915-016-0241-x

**Published:** 2016-03-23

**Authors:** Mustafa Khammash

**Affiliations:** Department of Biosystems Science and Engineering, ETH Zürich, Switzerland

## Abstract

In his splendid article “Can a biologist fix a radio? — or, what I learned while studying apoptosis,” Y. Lazebnik argues that when one uses the right tools, similarity between a biological system, like a signal transduction pathway, and an engineered system, like a radio, may not seem so superficial. Here I advance this idea by focusing on the notion of *robustness* as a unifying lens through which to view complexity in biological and engineered systems. I show that electronic amplifiers and gene expression circuits share remarkable similarities in their dynamics and robustness properties. I explore robustness features and limitations in biology and engineering and highlight the role of negative feedback in shaping both.

## Robust or fragile? A tale of two circuits

What exactly is to be understood by the term *robustness* in the context of a given system? A definition that is narrower in scope than the one given earlier is more useful. Indeed a more functional interpretation must include explicitly or implicitly the particular system property or phenotype whose robustness is being investigated, together with the specific adverse conditions or disturbances that it must withstand. In this sense, robustness is not so much an attribute of an entire system, as it is a property of some of its facets. It is instructive to explore this and other robustness issues using concrete examples, and so I shall investigate the robustness of two systems from very different disciplines: electrical engineering and biology.

In the early history of electronic technology, at no time was the need to achieve robustness more urgent than in the 1920s. At that time, transcontinental telephony required electronic amplifiers with high gain (amount of amplification) to boost telephone signals sufficiently for transmission over long distances. The use of vacuum tubes in the design provided the necessary high gain, but there was a problem. The vacuum tubes had uncertain and variable characteristics that introduced distortions and prevented the reliable prediction of the gain of the amplifier, which needed constant calibration. Harold Black, a Bell Labs engineer who worked on the problem, described it thus: “every hour on the hour — for 24 hours — somebody had to adjust the filament current to its correct value. In doing this, they were permitting plus or minus 1/2-to-1 decibel variation in amplifier gain, whereas for my purpose the gain had to be absolutely perfect.” In other words, the amplifier gain was not robust to the inevitable variations of the vacuum tube amplifier parameters. The problem resisted many attempts at its solution until 1927, when in a stroke of great insight, Black found a brilliant and simple solution. He realized that if he fed back a portion of the output of the amplifier into its input in a negative phase, the gain of the amplifier and its output should be reliably stabilized. Prototypes indeed demonstrated dramatic robustness of the amplifier output, noise attenuation, and far better overall performance. At the time, Black’s idea ran counter to accepted theory, and it took a full nine years for the patent office to award him a patent for his invention. Thus, the negative feedback amplifier was born — an invention that is considered by some to be the most important breakthrough of the 20th century in electronics.

Meanwhile, several billion years earlier, nature’s evolutionary explorations led to the discovery of negative feedback as a central strategy for regulating the internal cellular environment. The prevalence of negative feedback at every level of biological organization is a testament to this strategy’s effectiveness in achieving robust regulation of cellular processes and in successfully counteracting disturbances that act to push the system into disequilibrium. While biological systems and engineered ones may seem to be worlds apart due to their vastly different substrates, time-scales, and mechanistic implementation, I will show that they in fact have much in common. To make this point, I shall look more closely at two systems: the negative feedback amplifier and the autoregulatory gene expression circuit. Not only do they exhibit similar robustness and fragility properties, but the dynamic equations that describe them are nearly the same. I start by examining the robustness properties of the amplifier which will then help us understand those of the gene expression circuit. Prior knowledge of electronics is not required; readers unfamiliar with circuits can simply think of an amplifier as a dynamical system whose input-ouput behavior depends on a set of parameters.

### Robustness analysis of a feedback amplifier

An amplifier is an electronic device that receives as its input an electric signal (typically voltage), and delivers as its output an electric signal that is ideally a scaled replica of the input signal. This scaling is called the *gain* of the amplifier. If the gain is larger than one, the input will be amplified, which gives the device its name. Amplifiers are ubiquitous and can be found in our cell phones, computers, TV sets, radios, cameras, etc.

**Fig. 1.**
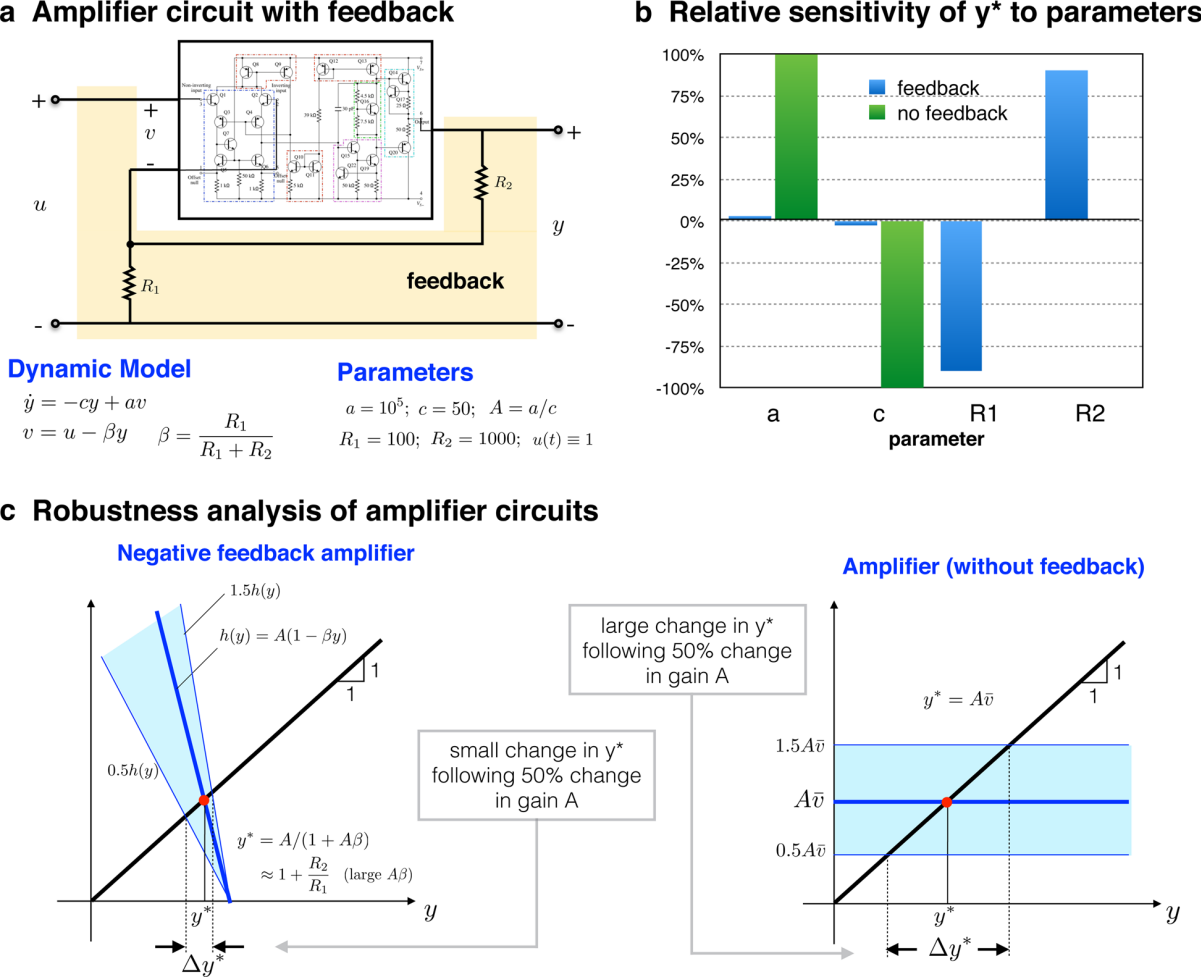
Robustness of two electronic operational amplifiers (with and without negative feedback). **a** A common model of a negative feedback amplifier with typical parameters. y is the output to input *u*(*t*), which I take to be unity. Shown also is the unregulated amplifier (circuit inside the rectangle) with input *v* and output *y*. This high gain amplifier is manufactured as an integrated transistor circuit. *a* and *c* are internal parameters such that *c* ^−1^ is the time constant and *A*=*a*/*c* is the amplifier gain. Negative feedback is introduced by adding the two resistors *R*_1_ and *R*_2_ in the configuration shown. The circuit is quite complex, but the simple first order model shown is a good representation of its behavior under typical operating conditions. The feedback resistors *R*_1_ and *R*_2_ are supplied by the user and are selected to tune the gain. **b** The robustness/fragility properties of the two amplifier circuits. For proper comparison, the input to the unregulated amplifier, $v(t)\equiv \overline {v}$, is chosen so that the corresponding output *y* ^∗^ matches that of the negative feedback amplifier. For the feedback amplifier, *y* ^∗^ is extremely robust to variations in the parameters *a* and *c*, in contrast to the unregulated amplifier. At the same time, *y* ^∗^ is quite sensitive to the values of the two resistors, underscoring its robust yet fragile character. **c** Graphical explanation of the difference in robustness properties of the two amplifiers. For both amplifiers, the abscissa of the point of intersection of the black line and the blue line gives *y* ^∗^. In the case of the feedback amplifier, the slope of the blue line is −*A* *β*. As *A* *β*>>1, one can see that *y* ^∗^ will be almost independent of *A*. Indeed, *y* ^∗^ depends almost exclusively on the ratio *R*_2_/*R*_1_, resulting in extreme robustness to *A*=*a*/*c*

Let me start, as Black did, with a high gain amplifier which does not employ negative feedback. I will denote its input voltage as *v* and its output voltage as *y*. During Black’s time, building such an amplifier required cumbersome vacuum tubes, but today such a device can be made with modern transistors. An electric circuit implementing one such amplifier is shown inside the rectangular box in Fig. 1a. One need not be concerned with the details of the internal circuitry (most users of the amplifier don’t know them anyway; they don’t need to!). Instead, I will focus on the relation between the input *v* and output *y*, which is particularly simple. Indeed, for slow time-scales the relation is a direct scaling of the input: *y*=*Av*, where *A* is the gain of amplifier. For faster time scales, a better model consists of a single first-order differential equation that more accurately captures the dynamics (see dynamic model in Fig. 1a). This equation is characterised by two parameters: *c*, the reciprocal of the time constant, which measures the amplifier’s speed of response, and *a*, which when divided by *c* gives the gain of amplifier, *A*.

The amplifier just described will suffer from many of the problems that faced Black in the 1920s, that is, high variability of the gain *A*. By adopting Black’s idea of including a version of the output in the input signal, one ends up with a negative feedback amplifier. This is straightforward to do: simply arrange that *v*=*u*−*βy*, where *β* is a constant parameter and *u* is a voltage signal that serves as the input to the feedback amplifier. This scenario is shown in the diagram shown Fig. 1a where the two resistors, *R*_1_ and *R*_2_, connecting the output to the input act to enforce *v*=*u*−*βy*. This is all that is needed to proceed with the robustness analysis of the negative feedback amplifier.

With negative feedback in place there are four model parameters, *a* and *c* for the amplifier (as explained above) and *R*_1_, *R*_2_ for the two resistors, i.e., ***θ***=(*a*,*c*,*R*_1_,*R*_2_), which are all positive. For simplicity, I shall take the input *u* to be constant (one) over time, and for a performance measure, I shall focus exclusively on the steady-state value of the output, *y*^∗^. Clearly, *y*^∗^ depends on the parameters ***θ***, so I can write *y*^∗^=*f*(***θ***) for some function *f*(·). With a performance measure at hand, one can ask when the system described by the above function may be considered to be ‘robust’. One possible answer is to equate system robustness with the ability of the output to withstand variations in all the model parameters. For example, one may want to insist that the property of interest, *y*^∗^=*f*(***θ***), is *insensitive* to variations in the four model parameters *θ*_1_,…,*θ*_4_ mentioned above. At this point, a quantitative measure of the sensitivity of *f*(***θ***) to each parameter *θ*_*i*_ is needed. I will use the following measure: the ratio of the relative change in the output *f*(***θ***) to the relative change in the parameter *θ*_*i*_ that caused it. I will refer to this (dimensionless) quantity as the relative sensitivity of the output to *θ*_*i*_, and I denote it by $S_{\theta _{i}}({\boldsymbol {\theta }})$. Mathematically,

$${\kern50pt}S_{\theta_{i}}({\boldsymbol{\theta}}) =\frac{\partial f ({\boldsymbol{\theta}})}{\partial \theta_{i}} \cdot \frac{{\boldsymbol{\theta}}_{i}}{f({\boldsymbol{\theta}})}. $$

While this expression of relative sensitivity evaluates the effect of small relative parameter changes, one could also evaluate the effect of larger parameter changes (such as a 100 *%* change or larger from the nominal value). This doesn’t alter any of the conclusions, however, so I will just use the above sensitivity expression.

Assigning typical values to the parameters ***θ*** (Fig. 1a), I can proceed with examining the relative sensitivity of *y*^∗^ to these four model parameters. Computing the relative sensitivity to parameter *a* in our example, one finds that *S*_*a*_(***θ***)≈0. Similarly, for the parameter *c*, *S*_*c*_(***θ***) is negligibly small. However, when one computes the relative sensitivities with respect to the remaining two parameters, one finds that $S_{R_{1}}({\boldsymbol {\theta }})\approx - 0.91$ and $S_{R_{2}}({\boldsymbol {\theta }})\approx 0.91$. In other words, a relative change in *R*_1_ or *R*_2_ results in a relative change in *y*^∗^ of almost the same magnitude (Fig. 1b). Sensitivity analysis thus informs us that while the variable of interest is insensitive to two of the parameters, it is quite sensitive to the remaining two. A similar conclusion can be reached were I to assess sensitivity to much larger changes in these parameters. Given this sensitivity, could one consider *y*^∗^ as a robust output of the system? More specifically, if these were the sensitivity properties of a system designed to keep *y*^∗^ constant, would one assess the performance of the system to be acceptable? As it turns out, the above circuit is that of a model 741 electronic operational amplifier [6] with typical parameter values (Fig. 1a). It is quite likely the most versatile electronic building block ever created! Every major integrated circuit manufacturer offers a version of it, and it can be found in a very large number of functioning electronic circuits. The success of this circuit is precisely due to the robustness of the output *y* to variations in parameters *a* and *c*, and one would be ill-advised to characterize the performance of the system as ‘non-robust’, even if the output is sensitively dependent on the parameters *R*_1_ and *R*_2_.

To get a deeper understanding of the issue, I will go back to the parameters of the model. The parameter a is directly related to the ‘open-loop gain’ of the amplifier, i.e., the ratio of the output to the input before any negative feedback is introduced. Such gain varies considerably, and could fluctuate up to several orders of magnitude. The introduction of negative feedback regulation introduces a dramatic improvement. This regulation is achieved by the two resistors *R*_1_ and *R*_2_. With these resistors in place, the amplifier gain (equal to *y*^∗^ here) is virtually insensitive to variations in *a* or *c*. Indeed, it can be shown (Fig. 1c) that *y*^∗^≈1+*R*_2_/*R*_1_, and is hence effectively independent of *a* and *c*. Instead, the gain is now heavily dependent on the values of the two resistors. It would appear that at the same time the feedback brought about robustness to parameters *a* and *c*, it introduced new fragilities, as can be seen in the strong dependence on the parameters *R*_1_ and *R*_2_. This is not a problem, however, as it is much easier to make precision resistors than precision unregulated high-gain amplifiers. Once the resistor values are selected, their values will remain virtually unchanged throughout their operation. In this way, the overall system is robust to variations in parameters that are *expected* to vary (e.g., *a*), but sensitive to parameters that can be expected to remain unchanged. This remarkably versatile system is thus *both robust and fragile*. It is robust to certain parameters but fragile in its strong dependence on others. Nor would one want the system to be robust to all parameters, as this would result in an amplifier whose gain cannot be tuned. The choice of resistors offers a simple and effective way to set the gain of the amplifier, a feature that cannot be realized had the output *y*^∗^ been insensitive to all the parameters. Such tradeoffs between robustness and fragility are common to virtually all complex engineered systems. Before I explain how negative feedback achieves this impressive feat, I will first bring in our biological example and compare it to the amplifier.

### Gene expression circuit

**Fig. 2.**
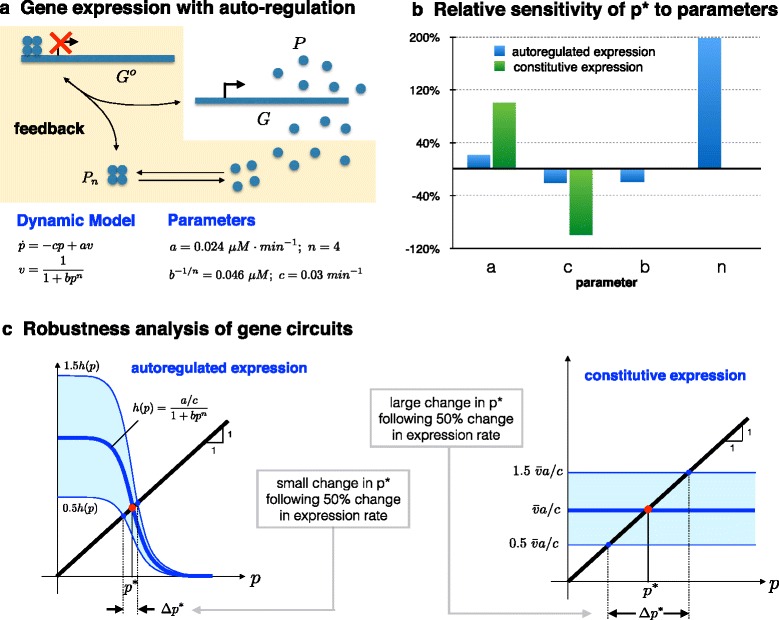
Robustness properties of two gene expression circuits (auto-regulated with negative feedback versus constitutively expressed without feedback). **a** The model of the auto-regulated gene expression circuit. Negative feedback is achieved by a Hill-type function resulting from the multimerization of the protein *P* into an n-mer *P*_*n*_, which in turn binds to the active gene *G* and represses it. Constitutive expression is modeled by an expression rate *av* that is independent of *p*. **b** The relative sensitivities of *p* ^∗^, the steady-state concentration of the protein, to the model parameters in both circuits. The auto-regulated circuit is robust to parameters *a* and *c*, in contrast to the constitutively expressed circuit, which is sensitive to both parameters. The auto-regulated gene circuit is, however, sensitive to parameter *n*. **c** A graphical explanation of the differences in robustness between both circuits. The intersection of the line and the graph of *h*(·) in the left figure (auto-regulated circuit) gives *p* ^∗^. Robustness in this circuit is achieved through high-gain and feedback, just as it is in the amplifier circuit. The higher the gain n the more robust the value of *p* ^∗^ will be to variations in the parameters *a*, *c*, and *b*. Indeed it can be shown that $S_{a}({\boldsymbol {\theta }}) \approx \frac {1}{n+1}$, $S_{c}({\boldsymbol {\theta }}) \approx \frac {-1}{n+1}$, $S_{b}({\boldsymbol {\theta }}) \approx \frac {-1}{n+1}$, and $S_{n}({\boldsymbol {\theta }}) \approx \frac {-n\log p^{\ast }}{n+1}$. In contrast, the constitutively expressed gene circuit lacks robustness to parameters a and c, even though it shares the same protein level *p* ^∗^ as the auto-regulated circuit. See also [20] for a general discussion of sensitivity of biochemical reactions and the effect of feedback

Here I explore the robustness properties of a simple gene expression circuit with autoregulation achieved through negative feedback. Negative autoregulation has been established as a network motif — one that appears, for instance, in the Escherichia coli transcriptional network far more frequently than would be expected in a random network [7]. Remarkably, the dynamics of an autoregulated gene expression circuit are very similar to those of the operational amplifier I discussed in the previous section, and many of the issues pertaining to robustness apply in a similar manner here as well. Figure 2a shows a simple circuit for gene expression and a corresponding dynamic model, describing the evolution of the expressed protein *p*. When the rate of gene expression, *v*, is independent of the protein level, this model corresponds to constitutive gene expression. On the other hand, when *v*(*t*) is dependent on *p* through a repression Hill function as shown in Fig. 2a, then the expression circuit is subject to negative feedback. It is interesting to note that if one were to linearize the nonlinear feedback term *v*(*t*) at the steady state value *p*^∗^, the dynamic equations for the gene expression circuit will be identical to those describing the operational amplifier. In fact, as can be seen in Fig. 2c, approximating the blue Hill function with a line near the intersection point will make the gene expression model identical to the amplifier model.

I shall now explore the sensitivity of the steady-state protein level, *p*^∗^, with respect to model parameters, both for the constitutive expression model and the model with negative feedback. In the constitutive expression case, the parameters are simply the expression rate *a* and the degradation rate *c*. In the feedback case, there are two additional parameters n and b that define the feedback repression Hill function (Fig. 2a). Specifically, *n* is the Hill coefficient, which determines the steepness of the Hill function, and *b* combines the association constant of protein *P* to form the *P*_*n*_ complex with the association constant of the resulting complex to DNA. One can get analytical expressions for the relative sensitivities of *p*^∗^ with respect to these parameters, and use them to study the robustness of the gene circuit. As can be seen in Fig. 2b, the constitutive expression model (no feedback) is quite sensitive to both *a* and *c*. For the typical nominal parameters ***θ*** shown in Fig. 2, the relative sensitivities to *a* and *c* are 1 and -1 respectively. In contrast, in the case of negative feedback, the relative sensitivity of *p*^∗^ to these two parameters is much smaller, namely *S*_*a*_(***θ***)≈0.22 and *S*_*c*_(***θ***)≈ −0.22. Similarly, *p*^∗^ shows small sensitivity to *b*, as can be seen from *S*_*b*_(***θ***)≈−0.2. At the same time, *p*^∗^ can be considerably sensitive to *n*. Indeed, it can be shown that *S*_*n*_(***θ***)≈2. A similar sensitivity dichotomy can also be seen in a stochastic model of this gene expression circuit.

How could one make sense of such robustness/fragility? We can begin to see the analogy with the negative feedback amplifier. Feedback of protein concentrations has ensured that the protein concentration output of the gene expression circuit will be relatively insensitive to the changing transcription and translation parameters. These parameters depend on many factors such as RNA polymerase levels, ribosome levels, etc., which in turn may depend on what other genes are active. These levels are also expected to be quite different from cell-to-cell. The robustness in these parameters comes at the cost of a new fragility manifested in the sensitive dependence of *p*^∗^ on *n*. However, this tradeoff appears well worth making, as *n* depends on the multimerization reaction of the protein *P* and is therefore not expected to vary much over time or among cells experiencing the same environment.

I should also point out that the robustness exhibited by both engineering and biological circuits is not restricted to variations in the parameters, but also applies to disturbances or stresses in the environment. Any extraneous voltage (or a load) at the output of the amplifier will be rejected, and the performance of the circuit will be unchanged. Similarly, an extra source of protein or a sink (e.g., loading due to nonspecific protein binding) will result in little change in the protein concentration, as the circuit acts to correct for such disturbances, particularly for high gain. The main message here is that both the operational amplifier circuit from electrical engineering and the gene expression circuit from biology share some key features, in spite of their vastly different substrates and time-scales. The output of interest in both circuits is robust to parameters that are expected to vary during the system’s operation, and sensitive to ones that experience little change. These features are characteristic of highly engineered (evolved) systems.

## Negative feedback: a robustness strategy with tradeoffs

Negative feedback is a key strategy for achieving robustness. For this reason, it has been studied extensively in the field of control engineering where the area of *robust control* has thrived since the late 70s. The effectiveness of this strategy can be seen in both the engineering and biological circuits considered thus far. Indeed, one can study the robustness of an unregulated operational amplifier (*R*_1_=0, $R_{2}=\infty $) or a constitutively expressed gene where negative feedback is absent. In each of these cases, even when the variable of interest is chosen to be identical to that observed when feedback is used, this variable will be vulnerable to variations in system parameters. Such parameters include the open-loop gain of the amplifier *A* or the transcription/translation rates for the gene expression circuit. In both instances, the robustness was brought about by the introduction of feedback. By trading off some gain, robustness to varying parameters is attained.

### How negative feedback brings about robustness

One can develop a clear understanding of how negative feedback brings about robustness by looking at the negative feedback amplifier in Fig. 1. For this circuit, one can compute the variable of interest explicitly. In particular, using the simple feedback amplifier model in Fig. 1a:

$${\kern50pt} y^{\ast} = \frac{A}{1+A\beta}\approx \frac{1}{\beta}, $$

where the last approximation is due to the high gain of the amplifier (*Aβ* is typically much larger than one). This shows clearly how negative feedback resulted in *y*^∗^ that is virtually independent of *A*=*a*/*c* and hence robust to variations in both *a* and *c*. Compare this to the unregulated open-loop amplifier (no feedback resistors, *β*=0) where:

$${\kern60pt} y^{\ast}= A {\overline{v}}. $$

Even when ${\overline {v}}$ is selected so that *y*^∗^ is the same for both amplifier configurations, the output *y*^∗^ (of the open-loop amplifier) will be far less robust to variations in *A*, and hence to variations in *a* and *c*. For robust operation, *A* must be very finely tuned. This is much more difficult to achieve in an amplifier than with resistors. Therefore, the feedback amplifier provides far superior robustness properties than the unregulated open-loop amplifier. In a very similar way, the regulated gene expression circuit offers better robustness properties than the constitutive gene circuit, especially when it comes to maintaining a steady value of *p*^∗^. Intriguingly, gene expression may be viewed as an amplifier that yields a large number of copies of a protein from a single copy of DNA. Autoregulation exchanges some of this high gain to achieve robustness to parameter variations. These parameters include transcription and translation rates and degradation rates. As in the electric amplifier, the autoregulated circuit is not without fragility. Indeed, as Fig. 2 shows, *p*^∗^ will be sensitive to variations in the Hill coefficient, *n*. However, *n* is not expected to change over the lifetime of the cell.

In both the amplifier and the gene expression circuit, the effect of high gain was considered only as far as it affects the steady-state performance of the two systems, both of which exhibited a constant equilibrium at steady-state. However, as we will see in what follows, the same idea applies in other regimes where external signals (e.g., disturbances) are time-varying and the system dynamics never reach a constant steady-state value. To see this, it is useful to understand how feedback attenuates disturbances. In feedback systems, the impact an external disturbance will have on an output of interest is often captured by the quantity |*S*|=|(1+*L*)^−1^|, where *L* is the so called ‘loop-gain’, i.e., the amplification a signal experiences after going once around the feedback loop. For the autoregulatory gene expression circuit, for example, *L* is the gain of the circuit if it were to be driven by an orthogonal transcription factor acting on a promotor of the same strength, but one which does not respond to the protein product. For the amplifier, *L*=*Aβ*.

With no feedback, *L* is zero, and the disturbance will not be attenuated; with high gain feedback, *L* is large, and the disturbance will be attenuated (small |*S*|). In practice *L* will not be the same for all signals. In particular, slowly varying signals will experience a different amplification going through the feedback loop than fast varying ones. The way one can study how a system responds to fast and slow signals is by evaluating its response to sinusoidal signals of different frequencies (number of cycles per second). This is because a slowly varying disturbance signal is made up of sinusoidal signals of low frequencies, while fast signals will also contain high frequency sinusoids. This makes *L* a frequency-dependent function, and for good disturbance rejection, it is only necessary that *L* be large at the frequencies of the disturbance. So if the system is to be immune to slowly varying disturbances, *L* needs to be large at low frequencies.

Based on the above discussion, achieving good attenuation of *constant* disturbances requires only that *L* be large at frequency zero (i.e., for constant signals). This is fortunate since achieving high gain at large frequencies is both difficult and fraught with other side effects. Intriguingly, achieving high gain at the zero frequency is not only possible, but it can be achieved perfectly, i.e., infinite gain is possible. This leads to perfect adaptation to constant disturbances. But what feedback dynamics can possibly offer infinite gain at zero frequency? The answer is *integral feedback*. This means that the signal that is fed back is first passed through an integrator, which integrates the signal with respect to time, thereby incorporating a measure of its past history into the feedback. Accordingly, the output of an integrator when the input (the integrand) is $\cos (\omega t)$ is given by $\omega ^{-1} \sin (\omega t)$, where *ω* is the frequency. This shows that the gain of the integrator is *ω*^−1^, which is indeed infinite at zero frequency. For this reason, integral feedback is a strategy that is very common in engineering and, as is becoming increasingly appreciated, also in biology. One well-studied example in biology where integral feedback has been implicated in robust perfect adaptation is bacterial chemotaxis [8, 9], in which the tumbling rate of a bacterium perfectly adapts to a change in nutrient concentration. This strategy allows bacteria to respond to a change in concentration of the nutrient regardless of the absolute concentration level. Other examples are calcium homeostasis [10] and yeast stress response [11].

### Robustness to environmental disturbances

**Fig. 3.**
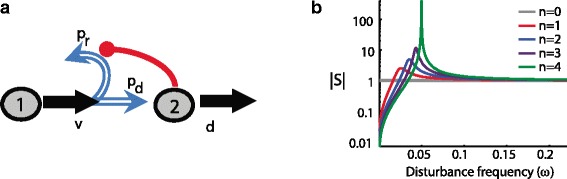
Renewal control. **a** A stem cell (type 1) can either regenerate or differentiate into a terminal post-mitotic cell (type 2). Negative feedback acts to affect the probability of regeneration. **b** The effect of sinusoidal variation in d (the disturbance) on |*S*|, the so-called ‘sensitivity function’, as a function of disturbance frequency. |*S*| is in turn related to the size of the corresponding fluctuation of the population of terminal cells (type 2). n reflects the strength of feedback, with stronger feedback resulting in better disturbance rejection (better robustness) at lower frequencies, at the price of amplifying the effect of disturbances at mid-frequencies (fragility)

I have argued that one way to maintain good performance in systems where some parameters are difficult to keep constant is to use negative feedback to trade off high gain with robustness to these parameters. Such a strategy can also be applied to achieve adaptation to time-varying environmental disturbances, whereby high gain at certain frequencies can be translated to rejection of disturbances at these frequencies. This could be demonstrated by introducing an external disturbance for our gene expression circuit — for example, another source for protein production (or degradation) — and then, depending on the disturbance frequency (e.g., if it is slowly or rapidly varying), showing that adaptation to this disturbance is achieved by the negative feedback circuit. Instead, however, I will look at another system: renewal control in stem cells [12]. Figure 3 shows the renewal control of a stem cell (type 1). The stem cell’s progeny can either be a stem cell (regeneration) or it could differentiate into a terminal, post-mitotic (type 2) cell. Feedback acts upon the stem cell to affect its probability of regeneration. A very simple model for this system can be written as:

(1) $${\kern50pt} \begin{aligned} \dot x_{1} &= (2p_{r}(x_{2})-1){vx}_{1} \\ \dot x_{2} &= 2p_{d}(x_{2}) {vx}_{1} -{dx}_{2},  \end{aligned}  $$

where *x*_1_ and *x*_2_ denote the concentration of stem cells and terminal cells, respectively, *v* is the cell-division rate, *p*_*d*_ is the probability that a daughter cell differentiates in a given division, *p*_*r*_ is the probability that a daughter remains a stem cell after division, and *d* is the probability that the terminal cell dies in a unit time. Negative feedback achieves renewal control due to the fact that *p*_*r*_ and *p*_*d*_ depend on *x*_2_ in such a way that *p*_*r*_(*x*_2_)+*p*_*d*_(*x*_2_)=1. For constant values of *p*_*r*_ and *p*_*d*_ (i.e., no feedback regulation), the trajectory of *x*_1_ blows up for *p*_*r*_>0.5 and tends to zero for *p*_*r*_<0.5, indicating that a robust nonzero steady state requires negative feedback. Not only does negative feedback bring about a stable nonzero steady-state value for *x*_1_ and *x*_2_, but it also achieves some robustness, as the concentration of terminal cells at steady state becomes dependent only on the relationship between *x*_2_ and *p*_*r*_ (the feedback term), and not on other system parameters [13]. This is reminiscent of both the feedback amplifier and the gene expression circuit.

To understand the response to dynamic external perturbations, it is necessary to specify a form for the feedback function *p*_*r*_(·). Following [12] I take $p_{r}(x_{2})=1/\left (\frac {3}{2}+\frac {1}{2}\left (\frac {x_{2}}{a}\right)^{n}\right)$. Here *n* reflects the strength of the feedback (feedback gain) and a is the value of *x*_2_ at which a balance of regeneration and differentiation is achieved. I can now evaluate how fluctuations of *d*, the rate of loss of terminally differentiated stem cell progeny, affect the terminal cell population *x*_2_. Such fluctuations occur because of injury, disease, or patterns of organ use [12]. A change in *d* that keeps it constant over time will have no effect on the steady-state population of the terminal cells. Moreover, the effect of slow fluctuations in *d* on the terminal cells population will be attenuated, and the higher the gain, n, the larger the attenuation. However, this robustness comes at a cost: the effect of fast changing fluctuations in *d* will not be attenuated, and may in fact be amplified. This is the fragility introduced by the same feedback that achieves robustness to slowly varying *d*.

The tradeoffs can be better appreciated by examining the effect of sinusoidal fluctuations in *d* on the size of the corresponding fluctuations in *x*_2_. This is captured in Fig. 3b. The horizontal axis measures the frequency *ω* of the sinusoidal fluctuations in *d*, while the vertical axis shows |*S*(*ω*)|, a quantity that is related to the effect of these fluctuations on the terminal cell concentration, *x*_2_. Note that lower frequency (slow) fluctuations are well attenuated, unlike those at higher frequencies. At mid-frequencies, the fluctuations are actually amplified. The stronger the feedback (higher *n*), the better the attenuation of lower frequency sinusoidal fluctuations, but the higher the amplification of mid-frequency fluctuations in *d*. This shows a robustness/fragility tradeoff that cannot be overcome with higher gain feedback. This also demonstrates a ‘conservation of robustness’. The more robustness is achieved at lower frequencies, the less robustness and more fragility is available at other frequencies. This conservation law has been studied extensively in the control literature [14, 15], and is characterized by a class of *Bode sensitivity integral* formulae of the form $\int _{0}^{\infty } \log |S(\omega)| \cdot f(\omega) d\omega = \text {constant}$, where the constant and *f*(·) are independent of *n*. In our particular example, it can be encapsulated as follows: no matter the feedback gain *n*, the area below the gray line |*S*|=1 is approximately equal to that above it (Fig. 3b). In other words, whenever robustness is realized (area below gray line), fragility is created elsewhere (area above gray line). Of course, as in the previous examples, a good system design ensures that the necessary fragility is arranged such that its effect is only seen when unexpected or unnatural disturbances are encountered, while robustness is achieved exactly when natural or common disturbances are encountered.

The frequency-dependent fragility that I just described appears routinely in man-made systems, but it has also been observed and reported in models of biological systems such as glycolysis [16] and cell lineage [12]. A recent research study [17] explored how yeast cells interpret environmental information that varies over time. The researchers examined cellular growth under various frequencies of oscillating osmotic stress and found that growth was in fact severely inhibited at a particular resonance frequency. They wrote “although this feature is critical for coping with natural challenges — like continually increasing osmolarity — it results in a tradeoff of fragility to non-natural oscillatory inputs...”. The authors aptly refer to this hyper-sensitivity as the Achilles’ heel of the yeast’s MAPK signaling network.

### Effect of topology on robustness-fragility tradeoffs

In the previous example, I showed that different feedback strengths lead to different tradeoffs of a conserved quantity (available robustness), but did not increase the amount of that quantity. An intriguing question is whether one can enhance overall robustness by changing the topology of the network being regulated. In this case, robustness may still be conserved for different feedback strengths, but the total amount that is conserved may possibly be increased. It turns out that this is indeed possible, and that some topologies are inherently more capable of delivering robustness than others. One example from engineering demonstrates this point clearly. It is well known that vehicle steering can be achieved either by turning the front wheels or the rear wheels of a vehicle. However, with the exception of slow vehicles like mobile cranes and forklift trucks, vehicles are almost universally steered using their front wheels. Why? The main reason is that vehicles that use rear-wheel steering exhibit what is called ‘non-minimum phase’ dynamics [18]. Systems with such dynamics respond to inputs in a non-intuitive way: they first respond in the opposite direction of the input before responding back in the expected direction. The reader would have noticed that when driving a car in reverse (analogous to rear-wheel steering in forward driving), turning the steering wheel in one direction leads the car to initially move slightly in the opposite direction before moving in the intended direction once its orientation has changed. A passenger sitting near the center of mass will feel an acceleration force that quickly switches direction after the initiation of a turn. The non-minimum phase dynamics make the car very difficult to steer at higher speeds, necessitating very slow movements (low bandwidth control) for stability. In contrast, when relegating the steering to the front wheels, the non-minimum phase dynamics disappear, and robust control of the vehicle is much easier to achieve. Before I show biological examples where these very same dynamics limit robustness, I will give one other engineering example where topological choices of a different nature place limits on achievable robustness.

**Fig. 4.**
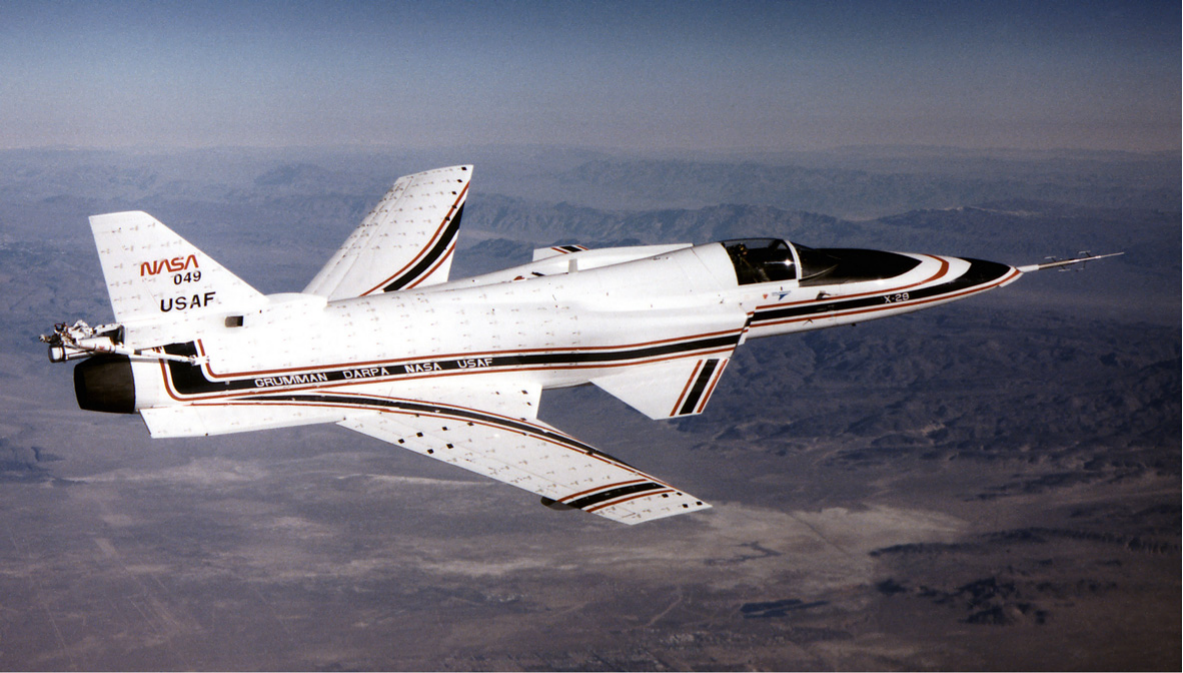
X-29 experimental aircraft. The forward-swept wings configuration of the X-29 makes the design of robust feedback control systems more difficult compared to more conventional aircraft. (Courtesy of NASA)

The X-29 experimental airplane shown in Fig. 4 was designed to have peculiar forward-swept wings and large canard surfaces for increased maneuvrability and aerodynamic efficiency. The designers knew that this configuration made the airplane dynamically unstable with no control, and that computer feedback regulation would therefore be essential for its operation. While the X-29 was indeed flyable using negative feedback regulation, it turned out (quite unexpectedly) that its robustness margins were too small to meet specifications. In control theory jargon, the available bandwidth was too small given the unstable dynamics [19]. The aircraft also exhibited non-minimum phase dynamics which conspired with the unstable dynamics to place substantial limitations on the achievable robustness. The problem could not be overcome with better feedback control systems. Indeed while engineers could select feedback controllers that increase robustness where it was needed at the expense of fragility elsewhere, the total available robustness to be eked out was limited. The limitations were severe enough that no acceptable control system was ever found, even though several design teams from different companies worked on the problem. The airplane was only allowed to fly due to special specification relief that was granted because it was an experimental airplane [19]. In retrospect, the choice of topology imposed a fundamental performance limitation that could not be overcome by feedback regulation. By choosing a different topology (e.g., less severe instability or even a stable airframe with traditional backward-swept wings), feedback designs that meet robustness specifications might easily be found. Though the robustness-fragility tradeoffs would still exist, good designs are much easier to achieve, and the best designs are far more robust than those achievable in the forward-swept aircraft like the X-29. In essence, the improved topology ensures that there is more overall robustness to be traded-off.

In biology, the effect of topology on the robustness availability can be used to assess different candidate models, and to favor some over others. It was exactly such considerations that led to a re-evaluation of the plausibility of the renewal control topology in Fig. 3. Indeed computational analysis of this topology showed poor robustness properties, including unfavorable disturbance rejection of periodic disturbances over certain frequency ranges. Dynamical analysis of this topology revealed the presence of non-minimum phase dynamics similar to those exhibited by a rear-wheel steered vehicle or the X-29. To see this, let us revisit the renewal dynamics modeled by Eq. 1 and consider the effect of a sudden increase in the renewal rate *p*_*r*_ on the concentration of terminal cells *x*_2_. Such an increase in the probability of stem cell renewal will immediately result in a reduction in the probability of differentiation of stem cells into terminal cells, causing *x*_2_ to start decreasing. However, the subsequent buildup in stem cell populations will lead to a gradual increase in the rate of differentiation, reversing the decreasing trend and leading to an ultimate increase in *x*_2_ — exactly the type of response reminiscent of non-minimum phase dynamics. As in the rear-wheel steering vehicle example, the unavoidable implication is that the topology has structural properties that can be expected to reduce achievable robustness.

**Fig. 5.**
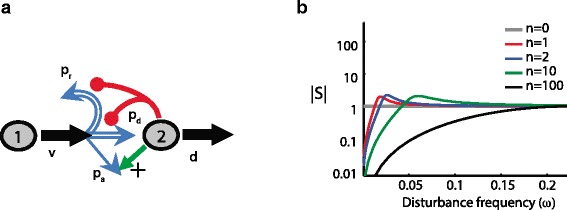
Fate control. **a** A stem cell (type 1) can either regenerate, or differentiate into a terminal post-mitotic cell (type 2), or have a third alternative fate that leads to a new branch. Negative feedback of the population of terminal cells acts on the probability of regeneration and differentiation, which necessarily leads to a positive feedback on the alternative fate as the three probabilities must sum to 1. **b** The effect of sinusoidal variation in *d* (the disturbance) on |*S*|, the ‘sensitivity function’, as a function of disturbance frequency. |*S*| is related to the size of the corresponding fluctuation of the population of terminal cells. *n* reflects the strength of feedback. For this fate control model *p*_*r*_(*x*_2_) is the same as in the renewal control case, while *p*_*d*_(*x*_2_) is taken to be *p*_*r*_(*x*_2_)/2. As in renewal control, stronger feedback results in better disturbance rejection (better robustness) at lower frequencies, at the price of poor disturbances rejection at mid-frequencies. Unlike renewal control, however, the system has significantly more capacity for disturbance rejection (more overall robustness), as indicated by the much larger area below the *gray line*

Since such non-minimum phase dynamics are attributable to the direct coupling between the probabilities of renewal and differentiation, one can alter the topology to rid it of these dynamics, leading to far superior robustness properties. One such topology, corresponding to a so-called fate control strategy, is realized by simply allowing lineage branching (Fig. 5), whereby stem cells can differentiate along a third trajectory, such as producing a different cell type, dying, or simply becoming quiescent. Examples of such branching exist during development and regeneration in various tissues. See [12] and the references therein.

In the fate control topology, the dynamics of the system reflect the fact that descendants of cell type 1, in addition to regulating the probability of stem cell regeneration and differentiation, also regulate the probability of production of a differentiated cell of a new type (Fig. 5). The dynamics of this new topology is given by:

$$\begin{array}{@{}rcl@{}} \dot x_{1} &=& (2p_{r}(x_{2})-1)vx_{1} \\ \dot x_{2} &=& 2p_{d}(x_{2}) vx_{1} -dx_{2}\\ \dot x_{3} &=& 2p_{a}(x_{2}) vx_{1} - d_{3} x_{3} \end{array} $$

where *p*_*a*_(*x*_2_) is the probability of choosing the new cell type fate. In this case, *p*_*r*_(*x*_2_)+*p*_*d*_(*x*_2_)+*p*_*a*_(*x*_2_)=1 holds. This means that negative regulation of *p*_*r*_(*x*_2_) need not imply a positive regulation of *p*_*d*_(*x*_2_), as their sum is no longer restricted to one. Instead simultaneous negative regulation of *p*_*r*_(*x*_2_) and *p*_*d*_(*x*_2_) is possible and in fact has superior robustness properties, as can be seen in Fig. 5. Indeed, using the same realization of *p*_*r*_(*x*_2_) as in renewal control, the fate control topology is much better at rejecting disturbances as well as other perturbations. This is reflected in the fact that the area under the grey line is considerably smaller than that for the renewal control in Fig. 3, and has a much smaller resonant peak, where fragility, and hence disturbance amplification, is at its maximum.

Another biological example that demonstrates a similar role of topology in enhancing the overall achievable robustness can be found in glycolysis [16]. In this autocatalyzed process, ATP feedback inhibits phosphofructokinase (PFK) reactions. Pyruvate kinase (PK) reactions are also known to be inhibited by ATP. Simple models of glycolysis that include only the PFK feedback but neglect the PK feedback can exhibit unstable as well as non-minimum phase dynamics. Just like in the X-29, this places severe limitations on the achievable robustness. Regardless of the PFK feedback strategy used, the system will have too much fragility. By simply bringing in PK feedback, these unfavorable robustness tradeoffs disappear, and the resulting topology will have far better dynamic performance.

## Concluding remarks

In advanced engineering systems as well as in biological systems, robustness is a property of a specific functionality or performance measure. When present, it indicates that the relevant function is relatively immune to certain perturbations, such as variations of system parameters or external disturbances that are expected to occur during the system’s lifetime. While this robustness is desirable, it is often not possible for a function or a performance measure to be robust to variations in all possible system parameters or to all perturbations. To be sure, optimized systems, whether engineered or evolved, are often sensitive to specific perturbations. But this typically does not pose any severe drawbacks, as these perturbations are not expected to be encountered frequently in the life of the system. This robust yet fragile character of such systems has implications for modeling and parameter inference. Naturally, when a measured variable is robust to some parameters, one expects that inferring the parameter from measurements of the variable is challenging, leading to practical lack of identifiability of these parameters.

I have argued that one way to achieve robustness to a set of parameters or disturbances is to use negative feedback. This allows the tradeoff of high gain with robustness to parameter variations or external disturbances. I have demonstrated these tradeoffs for constant disturbances at steady-state values of the output of interest, as well as for time varying disturbances, where rejection of disturbances at some frequencies can be effectively achieved at the expense of poor disturbance rejection at other frequencies. Such are the robustness tradeoffs of feedback, which can be encapsulated in quantitative conservation laws. The compelling aspect of such tradeoffs is their universality. As we have seen in this article, they apply to feedback systems regardless of their substrate and specific implementation details. They are the conservation laws of robustness that natural and man-made systems alike must obey.
